# Longitudinal Change in Appearance-Related Social Media Consciousness and Depressive Symptoms: A Within-Person Analysis during Early-to-Middle Adolescence

**DOI:** 10.1007/s10964-024-01998-5

**Published:** 2024-05-24

**Authors:** Anne J. Maheux, Jean-Philippe Laurenceau, Savannah R. Roberts, Jacqueline Nesi, Laura Widman, Sophia Choukas-Bradley

**Affiliations:** 1 Department of Psychology and Neuroscience, University of North Carolina at Chapel Hill, Chapel Hill, NC USA; 2 Department of Psychological and Brain Sciences, University of Delaware, Newark, DE USA; 3 Department of Psychology, University of Pittsburgh, Pittsburgh, PA USA; 4 Department of Psychiatry and Human Behavior, Warren Alpert Medical School of Brown University, Providence, RI USA; 5 Bradley Hasbro Research Center, Rhode Island Hospital, Providence, RI USA; 6 Department of Psychology, North Carolina State University, Raleigh, NC USA

**Keywords:** Adolescence, Social media, Body image, Depressive symptoms, Self-objectification, Online appearance concerns

## Abstract

Online appearance preoccupation may put adolescents at risk of developing mental health challenges, perhaps especially during early-to-middle adolescence. A random intercept cross-lagged panel model assessed within-person associations between *appearance-related social media consciousness* and depressive symptoms over three time-points with three months between waves. The sample (*n* = 1594) included U.S. adolescents aged 11–15 (*M*age = 13; 47% girls, 46% boys, 7% another gender; 37% Latine, 33% White, 18% Black, 7% Asian). Within-person increases in appearance-related social media consciousness were associated with subsequent increases in depressive symptoms, but not vice versa. There was no evidence of gender differences and results were robust to controlling for both time on social media and offline self-objectification. Thus, online appearance concerns precede mental health challenges during early and middle adolescence.

## Introduction

Social media use is nearly ubiquitous among U.S. adolescents, and youth are obtaining smartphones and social media accounts at increasingly younger ages (Rideout et al., 2022). Systematic reviews and meta-analyses indicate that the association between time on social media and depressive symptoms among adolescents is inconsistent (see Orben, 2020), leading to the consensus that research must instead address specific online behaviors and subjective cognitive and emotional experiences (Odgers & Jensen, 2020). Many of the most popular social media platforms, such as Snapchat, Instagram, and TikTok, are highly visual and may encourage appearance concerns (Rodgers & Melioli, 2016), such as *appearance-related social media consciousness*, which is a persistent preoccupation with appearing physically attractive online (Choukas-Bradley et al., 2019). Critically, developmental science addresses how individuals change over the life course and thus requires longitudinal designs that can isolate within-person change. To date, research on online appearance preoccupation has relied on mostly cross-sectional, between-person designs (see Choukas-Bradley et al., 2022) and focused on late adolescence and early adulthood, despite recent evidence that young adolescents may be at heightened risk for negative effects of digital media use (Orben et al., 2022). Thus, associations between individual-level change in social media appearance concerns and depressive symptoms among young adolescents remain unclear. The current study examines longitudinal within-person associations between appearance-related social media consciousness and depressive symptoms during early-to-middle adolescence.

### Adolescence and the Development of Depressive Symptoms

During early and middle adolescence (approximately ages 10–13 and 14–17, respectively), youth undergo immense cognitive, biological, and social changes associated with pubertal development (Rogol et al., 2002). Adolescent development is marked by the centrality of peer approval and status, identity exploration and cognitive development, initiation of romantic and sexual relationships, and heightened self-consciousness (Dahl et al., 2018). Biological changes during puberty put adolescents at risk of heightened body image concerns (Markey, 2010), which are linked with later depressive symptoms, especially among girls (Sharpe et al., 2018). Neurobiological and cognitive changes in adolescence lead to heightened sensitivity to information related to oneself and the social world (Crone & Dahl, 2012), which may intensify the link between interpersonal stressors and depressive symptoms (Rudolph et al., 2008). Adolescents also experience an egocentric preoccupation with how others perceive their appearance and behavior—i.e., *imaginary audience* ideation (Galanaki, 2012).

These normative developmental changes put adolescents at risk of negative body image perceptions and mental health problems (Maughan et al., 2013). During adolescence, attractiveness is highly correlated with popularity, especially for girls (Mayeux & Kleiser, 2020), frequently discussed among peer groups (Jones et al., 2004), and relevant for dating relationships (Ha et al., 2010). Objectification theory highlights how exposure to idealized images in traditional media manifests in heightened attention to one’s appearance, a tendency to imagine the self from an outsider’s perspective (self-objectification), and ultimately body shame and mental health problems (Fredrickson & Roberts, 1997). The normative cognitive, biological, and social features of adolescent development exacerbate the tendency to self-objectify and, ultimately, experience body shame and depressive symptoms, especially for girls (Daniels et al., 2020). Cognitive theories of depression contend that distorted, overly negative thinking contributes to depressive symptoms (Beck, 2002), whereas interpersonal theories state that depressed individuals often display interpersonal deficits and disruptions, such as excessive reassurance-seeking and rejection (Rudolph et al., 2008). Body image concerns reflect both cognitive evaluations of one’s appearance, as well as social evaluations of how the self appears to others, and are transdiagnostic risk factors for multiple mental health problems, including depressive symptoms (Sharpe et al., 2018) and disordered eating behaviors (Ferreiro et al., 2014), especially among girls.

Developmental vulnerabilities to depressive symptoms may be exacerbated in the context of social media. Rates of depression have been steadily increasing among adolescents, especially girls (Geiger & Davis, 2019), and some have posited that these trends are linked with the advent of new technologies (Twenge et al., 2018). Importantly, mixed results and small effect sizes have led researchers to a consensus that the specific behaviors and subjective experiences youth have online are more relevant for mental health than is time spent on social media (Odgers & Jensen, 2020). Social media provides unprecedented opportunities for adolescents to engage in distorted cognitions or maladaptive interpersonal behaviors, such as drawing negative conclusions from ambiguous online interactions (Özparlak & Karakaya, 2022), attending only to negative self-referential online content (Ridolfi et al., 2011), fixating on their physical appearance in social media photos (Nesi et al., 2021), and seeking reassurance through quantifiable metrics of peer approval (Nesi & Prinstein, 2015). Given the links between these forms of social media use, negative body image outcomes, and depressive symptoms (Choukas-Bradley et al., 2022), more work is necessary to understand how specific appearance-focused social media experiences may put adolescents at risk for depression.

### Appearance-Related Social Media Consciousness and Adolescent Development

Certain features of social media encourage preoccupation with one’s appearance. Content on social media platforms is highly visual, permanent, and public, such that adolescents have constant access to photos of themselves and their peers (Nesi et al., 2018). The “24/7” availability of social media makes online interactions possible at all times, creating more opportunities—but also potentially greater pressure—for adolescents to engage with or be visible to peers (Nesi et al., 2018). Self-objectification in the context of social media may occur when adolescents are exposed to idealized images of peers on social media (Slater & Tiggemann, 2015) or engage in selfie-related behaviors (e.g., selfie editing; Terán et al., 2020). Self-objectification may even occur during offline moments when photos or videos could be taken and posted online (Choukas-Bradley et al., 2020).

Appearance-related social media consciousness is the persistent concern about looking attractive on social media (Choukas-Bradley et al., 2019). Appearance-related social media consciousness includes imagining how one’s photos look to a social media audience, overvaluing physical attractiveness online, and carefully editing one’s photos (Choukas-Bradley et al., 2020). Among U.S. high school students (i.e., ages 14–19), appearance-related social media consciousness is cross-sectionally associated with depressive symptoms above and beyond both time on social media *and* body surveillance, the behavioral manifestation of self-objectification (Choukas-Bradley et al., 2020). These results highlight the importance of not only differentiating appearance-related social media consciousness from “screen time,” but also distinguishing between online and offline appearance cognitions. To date, only one published longitudinal study has explored this association, finding that, among U.S. high school students (ages 15–19), higher appearance-related social media consciousness at baseline was associated with increased depressive symptoms one year later (Maheux et al., 2022a). In other words, youth who are higher in appearance-related social media consciousness *relative to their peers* report increases in depressive symptoms, yet it remains unclear how experiencing heightened appearance-related social media consciousness *relative to one’s prior level of appearance-related social media consciousness* may drive increased depressive symptoms. Only within-person analyses of repeated measurements can identify how individual-level change unfolds across development (Curran & Bauer, 2011). Some research has suggested that increased posting of appearance-focused content on social media at the within-person level is associated with within-person decreases in body image (Schreurs & Vandenbosch, 2022), whereas other work shows that within-person increases in liking and commenting on others’ social media posts are associated with within-person decreases in appearance self-esteem (Steinsbekk et al., 2021). These prior studies provide insight into developmental change processes—individuals engaging in appearance-related social media experiences *more than they usually do* may drive body image experiences. However, no prior research has addressed within-person change processes in the co-development of appearance-related social media use and depressive symptoms, including among potentially vulnerable young adolescents.

Gender differences in associations between appearance-related social media consciousness and depressive symptoms also warrant more investigation. The differential susceptibility to media effects model highlights how individual and contextual factors shape adolescents’ social media experiences (Valkenburg & Peter, 2013). Cross-sectional research consistently finds that, relative to boys, girls report more preoccupation with their online appearance, but that the association between appearance-focused social media experiences and depressive symptoms is similar for boys and girls (e.g., Hawes et al., 2020). Thus, although girls may be predisposed to heightened online appearance concerns, the risk of negative outcomes related to such concerns may be similar across gender. However, these prior studies were conducted with middle adolescents (e.g., mean age of 15–16, Choukas-Bradley et al., 2020) or older adolescents (e.g., mean age of 19 for young adult sample, Hawes et al., 2020). Research on developmental sensitivity to social media suggests differences relating to sex differences in pubertal development, with boys most sensitive during middle adolescence (14–15) and girls most sensitive during early adolescence (11–13; Orben et al., 2022). Thus, more research is needed exploring if gender differences emerge among early-to-middle adolescents.

## Current Study

Higher appearance-related social media consciousness has been linked to increased risk for depressive symptoms in middle to late adolescence at the between-person level, but a rigorous investigation of longitudinal associations in early adolescence is needed. The current study examined within-person associations between appearance-related social media consciousness and depressive symptoms among U.S. early-to-middle adolescents over one academic year and explored gender differences in this association. It was hypothesized that within-person changes in appearance-related social media consciousness would be associated with subsequent within-person changes in depressive symptoms. Comparisons across gender were exploratory given the paucity of prior work exploring gender differences in these longitudinal associations among early-to-middle adolescents.

## Methods

### Participants and Procedure

Data were collected from U.S. 8^th^ grade students at three waves during the 2020–2021 academic year (October, January, March). At baseline, participants (*n* = 1594) ranged in age from 11 to 15 (*M* = 13.15, *SD* = 0.48; 77% 13-year-olds, 17% 14-year-olds; 4% 12-year-olds). The sample included 47.49% girls, 46.05% boys, 3.63% with another gender identity, and 2.82% missing gender identity information. The sample was racially/ethnically diverse (36.76% Hispanic/Latine, 32.37% White non-Hispanic, 17.50% Black, 7.21% Asian, and 6.15% multiracial or another race/ethnicity) and 40.21% received free or reduced-price lunch.

Recruitment and data collection were conducted by the Character Lab Research Network (CLRN), a consortium of schools and researchers collaboratively collecting school-based data. Participants were recruited from a single school district in Florida (U.S.) across 21 school locations (sample sizes per school ranging from *n* = 1 to *n* = 256). Variance in appearance-related social media consciousness (ICC = 0.014) and depressive symptoms (ICC = 0.006) was almost entirely between people (rather than between schools), and thus school was not considered in analyses. CLRN is designated a School Official under FERPA and all CLRN studies comply with the U.S. Protection of Pupil Rights Amendment (PPRA), guidelines that restrict researchers from asking questions considered sensitive. Parents are informed of CLRN research procedures and adolescent participants provide assent prior to participation, during which they are informed that they can withdraw from the questionnaire at any time with no penalty.

On the day of data collection, students completed online surveys via Qualtrics during classroom periods. The final sample size was determined by the CLRN protocol and students who were available and provided assent on the days of data collection. All participants recruited into the study (*n* = 1615) were included in analyses, with the exception of those who were not in 8^th^ grade (*n* = 2) and those who were missing data for appearance-related social media consciousness and depressive symptoms at all time-points (*n* = 19), yielding a final sample of 1594. Those ultimately not included in analyses were no different than those included in terms of gender, age, or race/ethnicity, but were more likely to report free or reduced-price lunch eligibility, χ^2^(1) = 3.23, *p* = 0.02. All participants reported hybrid instruction during the 2020–2021 year. All procedures were approved by the CLRN external IRB (Advarra) and university Institutional Review Board. Study design and methods for the overarching studies were preregistered here: https://osf.io/n6vb4 and materials needed to reproduce this study are available here: https://osf.io/d2qt9/.

### Measures

#### Demographics

Participants self-reported their gender at each wave based on a single item dictated by PPRA guidelines. Response options included “girl,” “boy,” “other,” and “prefer not to say.” Participants were categorized as “another gender identity” if they indicated “other” or “prefer not to say” at any wave, or if they indicated “girl” at one wave and “boy” at another wave. Participants’ eligibility for free or reduced-price lunch, a proxy for lower SES, and race/ethnicity were collected from schools.

#### Appearance-related social media consciousness (ASMC)

The ASMC Scale was administered at each wave (Choukas-Bradley et al., 2020). The 13-item scale, developed for adolescents, has strong internal consistency, test-retest reliability, and convergent validity (Choukas-Bradley et al., 2020). Respondents reported the frequency of thoughts and behaviors related to ongoing awareness of one’s appearance on social media (e.g., “During the day, I spend time thinking about how attractive I might look when people see pictures of me on social media”; “I look at pictures of myself on social media again and again”) on highly-visual social media platforms (e.g., Facebook, Snapchat, Instagram), on a 7-point scale (1 = *Never* to 7 = *Always*). Items were averaged. Internal consistency was excellent (T1 α = 0.94; T2 α = 0.95; T3 α = 0.96).

#### Depressive symptoms

Symptoms of depression in the past two weeks were assessed at each wave with the 13-item Short Mood and Feelings Questionnaire (SMFQ; Sharp et al., 2006), validated among adolescents (Turner et al., 2014). Participants indicated agreement with statements (e.g., “I felt miserable or unhappy”) on a scale from 0 = *not true* to 2 = *true*. Items were summed. Internal consistency was excellent (T1 α = 0.91; T2 α = 0.92; T3 α = 0.92).

#### Covariate: time on social media

Participants responded to a single item at each assessment to indicate if they have ever used social media (examples were provided of Instagram, TikTok, Snapchat, or Facebook). Those who responded “yes” were shown the following question to assess time on social media: “On average on a typical day, how much TIME do you spend using ANY social media?” Social media was defined for participants as “any apps or websites that involve social interaction, such as Instagram, Snapchat, or Facebook.” Responses ranged from 0 = *Less than 1* *hour* to 10 = *10 or more hours* per day. This measure of time on social media has been used before with adolescents (Choukas-Bradley et al., 2020).

#### Covariate: self-objectification

Participants responded to 13 items from the 14-item Self-Objectification Beliefs and Behaviors Scale (SOBBS; Lindner & Tantleff-Dunn, 2017), for which participants indicate agreement with statements related to internalizing an observer’s gaze (e.g., “I often think about how my body must look to others”) and believing that one’s body can represent oneself (e.g., “How I look is more important to me than how I think or feel”). One question about sexual attraction was removed to be compliant with school data collection policies. Responses ranged from 1 = *strongly disagree* to 5 = *strongly agree*. Items were averaged. Internal consistency was strong (T1 α = 0.94; T2 α = 0.95; T3 α = 0.95).

### Data Analytic Plan

Descriptive statistics and bivariate correlations were derived and variable distributions were visualized in R version 3.6.1 (R Core Team, 2021). The depressive symptoms variables were highly skewed. Random intercept cross-lagged panel models (RICLPMs) are not currently available for highly skewed (e.g., count) data; thus, the variables were transformed into a distribution appropriate for the assumptions of maximum likelihood estimation (i.e., that variables are normally distributed; see Bollen, 1989). Specifically, quantiles were derived from the depressive symptoms data at T1 (thresholds at values of 0, 2, 5, 9, 26); these thresholds were used to create five-category ordinal variables for the depressive symptom scores at each time point (see DeCoster et al., 2011). Maximum likelihood estimation with robust standard errors (MLR) was used to account for non-normality in the ordinal variables and full information maximum likelihood (FIML) was used to address missing data.

**Fig. 1:**
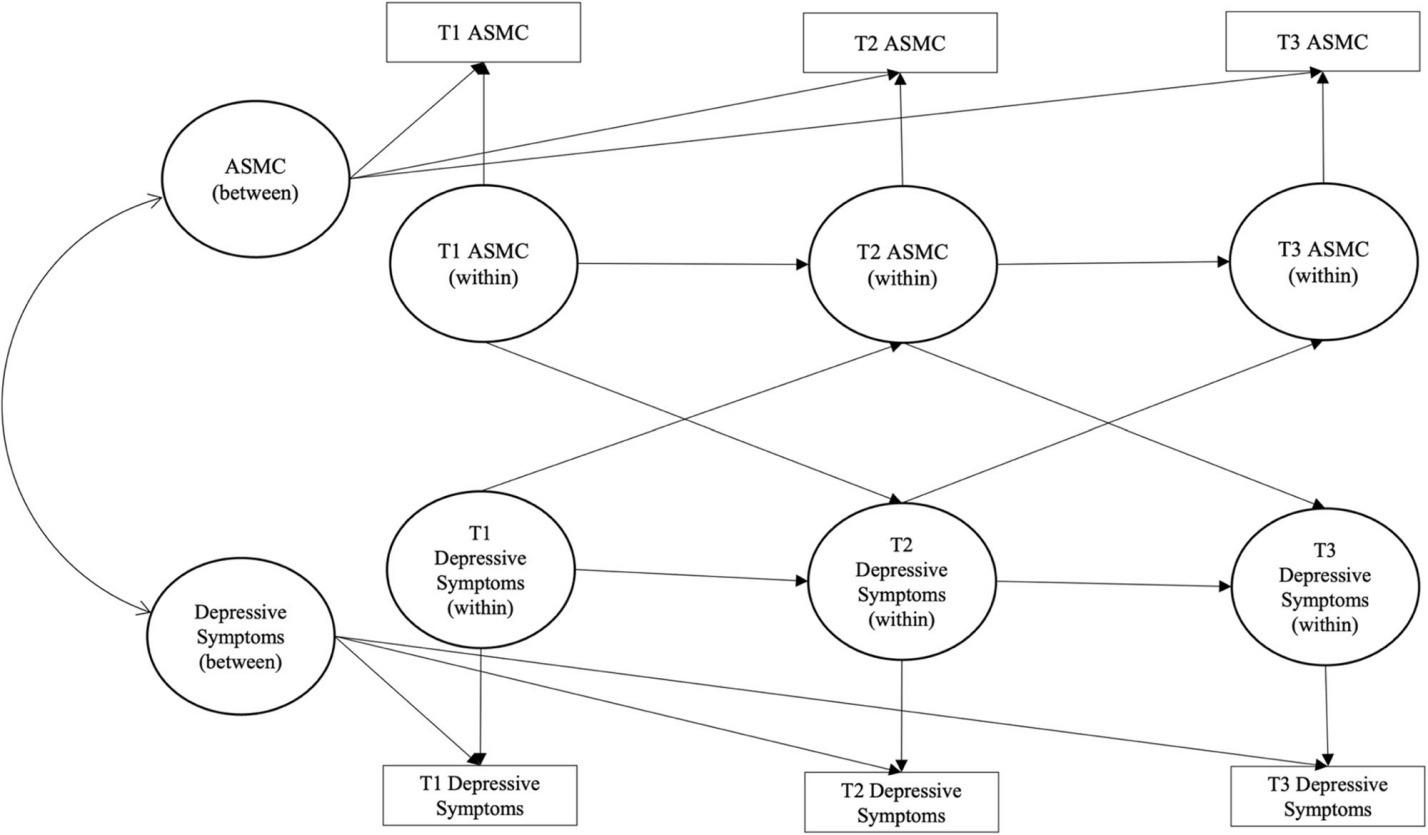
Conceptual random intercept cross-lagged panel model of between-person and within-person associations between appearance-related social media consciousness (ASMC) and depressive symptoms. Note. For parsimony, residual covariances between endogenous latent within-person factors and covariance between exogenous latent within-person factors are not shown.

A random intercept cross-lagged panel model (RICLPM) was constructed with appearance-related social media consciousness (ASMC) and depressive symptoms as between-person and within-person factors, with autoregressive and cross-lagged paths freely estimated between subsequent time-points (see Hamaker et al., 2015). Figure 1 presents a conceptual model of the RICLPM. Separate models in which the autoregressive parameters, and subsequently the cross-lagged parameters, were constrained to equality across time were compared to the baseline, unconstrained model. Chi-square difference tests using a sandwich estimator were used with MLR estimation to address potential violations of assumptions of normality and homoscedasticity when comparing fit between models (Satorra & Bentler, 2001). Constraints on the parameters across time (i.e., the autoregressive and cross-lagged within-person paths) that did not worsen fit relative to the freely estimated model were retained for parsimony.

Finally, multiple-group models were constructed in which all parameters were free to vary across gender. This baseline multiple-group model was compared to a model in which the cross-lagged paths were constrained to be equal across groups using the same robust chi-square difference test (Satorra & Bentler, 2001). Non-significant nested comparisons indicate that cross-lagged paths can be constrained to equality without impairing overall model fit, and thus are not significantly different across groups (Mulder & Hamaker, 2021). The gender comparison analyses included only adolescents who identified as boy or girl.

Several sensitivity analyses were conducted to address the robustness of results. First, two models were constructed to examine the role of covariates: time on social media and self-objectification. One model was constructed separately for each covariate. Using the final model specification determined from the original model (i.e., without covariates), these models included T1, T2, and T3 measures of the covariate as both between-person and within-person factors, to control for both between-person stability and within-person change in time on social media and self-objectification, respectively. Next, given that the depressive symptoms variable was transformed into an ordinal variable, the final RICLPM was constructed with depressive symptoms identified as a categorical variable in Mplus version 8 (Muthén & Muthén, 2017), following recent recommendations for cross-lagged panel models using ordinal data (Muthén, 2022; i.e., using robust weighted least squares mean and variance adjusted estimation [WLSMV] with theta parameterization and setting residual variances of observed indicator variables equal to 1). Additionally, given that some participants indicated never using social media, and thus may have had limited or different opportunities to experience ASMC, the final RICLPM was conducted with only participants who reported ever using social media (*n* = 1488). Finally, the final model was re-run with only participants with data at all waves (*n* = 874) to explore if results were robust to FIML procedures for missing data.

## Results

### Descriptive Results

Descriptive statistics and bivariate correlations for all study variables are shown in Table 1. Girls reported higher ASMC and depressive symptoms than boys.

**Table 1:** Bivariate correlations among study variables

	Full sample *n* = 1594 *M* (*SD*)	Girls *n* = 757 *M* (*SD*)	Boys *n* = 734 *M* (*SD*)	1.	2.	3.	4.	5.	6.
1. ASMC T1	2.54 (1.39)	3.07 (1.45)	1.99 (1.08)	–	0.64	0.49	0.36	0.32	0.30
2. ASMC T2	2.41 (1.37)	2.87 (1.43)	1.86 (1.03)	0.80	–	0.63	0.27	0.33	0.25
3. ASMC T3	2.41 (1.40)	2.83 (1.46)	1.89 (1.09)	0.70	0.84	–	0.16	0.19	0.34
4. Depressive Symptoms T1	3.12 (1.33)	3.47 (1.30)	2.71 (1.24)	0.51	0.44	0.33	–	0.64	0.50
5. Depressive Symptoms T2	2.99 (1.36)	3.33 (1.33)	2.56 (1.25)	0.45	0.50	0.41	0.75	–	0.64
6. Depressive Symptoms T3	2.82 (1.42)	3.12 (1.43)	2.36 (1.27)	0.40	0.46	0.46	0.59	0.70	–

Means and standard deviations are derived from mean scale scores for ASMC (range: 1–7) and mean scale scores for the transformed version of the depressive symptoms variable (range: 1–5). Girls’ mean scores on all variables are significantly higher than boys’ scores at *p* < 0.001. Girls’ correlations are presented below the diagonal; boys’ above. All correlations significant at *p* < 0.001

*T1* Time 1; *T2* Time 2; *T3* Time 3; *ASMC* appearance-related social media consciousness

### Random Intercept Cross-Lagged Panel Model

Nested model comparisons suggested that constraining the autoregressive paths, Δχ^2^(2) = 5.08, *p* = 0.079, and the cross-lagged paths, Δχ^2^(2) = 0.43, *p* = 0.807, resulted in no significant decrements in fit. Therefore, in the final model, autoregressive and cross-lagged paths were constrained to equality over time. The final model fit the data well, χ^2^(5) = 10.64, *p* = 0.06, *RMSEA* = 0.027, 95% CI *RMSEA* [0.00, 0.049], *SRMR* = 0.02, *CFI* = 1.00.

In the final model, the random intercepts of ASMC and depressive symptoms (i.e., the stable, between-person components) were positively associated: *r* = 0.51, *b* = 0.47, *se* = 0.19, *p* = 0.012. Within-person change in ASMC was associated with subsequent within-person change in depressive symptoms (i.e., the within-person cross-lags): *b* = 0.25, *se* = 0.08, *p* = 0.002. There was no evidence for the opposite direction of effects: within-person change in depressive symptoms was not significantly associated with subsequent within-person change in ASMC: *b* = 0.10, *se* = 0.06, *p* = 0.113. See Table 2 for full within-person model results.

**Table 2:** Within-person autoregressive and cross-lagged paths and within-wave covariances from the random intercept cross-lagged panel model

	Full sample (*n* = 1594)			
	*b*	*SE*	β	*p*
Autoregressive (lagged) paths				
ASMC → ASMC	0.606	0.120	0.580, 0.610	<0.001
DS → DS	0.391	0.088	0.365	<0.001
Cross-lagged paths				
ASMC → DS	0.246	0.079	0.241, 0.247	0.002
DS → ASMC	0.096	0.060	0.085, 0.092	0.113
Within-person within-wave covariances				
ASMC T1 with DS T1	0.444	0.181	0.482	0.014
ASMC T2 with DS T2	0.212	0.040	0.310	<0.001
ASMC T3 with DS T3	0.202	0.048	0.289	<0.001

Cross-lagged and autoregressive paths are constrained to be equal across time. Some standardized parameters vary over time for constrained paths due to different variances.

*ASMC* appearance-related social media consciousness; *DS* depressive symptoms

### Gender Comparison

A multiple group model in which all parameters were free to vary across gender (i.e., boys vs. girls; autoregressive and cross-lagged paths were constrained across time as determined in the single-group model) was compared to a nested model in which all cross-lagged parameters were constrained across groups. Constraining the cross-lagged paths resulted in no significant decrement in model fit, Δχ^2^(2) = 1.38, *p* = 0.501. Thus, there was no evidence that the within-person associations between change in ASMC and change in depressive symptoms differed across boys and girls.

### Sensitivity Analyses: Covariates

Two models were constructed to test the robustness of the results in the context of controlling for covariates. First, time on social media was included in the final RICLPM model as both between- and within-person factors. Full model results are presented in Supplementary Table 1. The same pattern of results was found for the cross-lagged paths; within-person change in ASMC was associated with subsequent within-person change in depressive symptoms (*b* = 0.24, *se* = 0.08, β = 0.23–0.24, *p* = 0.003), but not vice versa (*b* = 0.10, *se* = 0.06, β = 0.09–0.10, *p* = 0.108). Notably, within-person change in time on social media was not associated with prior or subsequent change in either ASMC or depressive symptoms (*p*s = 0.097–0.466). However, the random intercepts for ASMC and time on social media were significantly positively correlated (*r* = 0.37, *b* = 0.66, *se* = 0.28, *p* = 0.019), but the random intercepts for depressive symptoms and time on social media were not (*r* = 0.22, *b* = 0.39, *se* = 0.26, *p* = 0.128).

Second, self-objectification was included in the final RICLPM model as both between- and within-person factors. Full model results are presented in Supplementary Table 2. Again, the same pattern of results was found for the original cross-lagged paths; within-person change in ASMC was associated with subsequent within-person change in depressive symptoms (*b* = 0.22, *se* = 0.09, β = 0.21–0.22, *p* = 0.011), but not vice versa (*b* = 0.10, *se* = 0.06, β = 0.09–0.10, *p* = 0.111). In this model, within-person change in ASMC was associated with subsequent within-person change in self-objectification (*b* = 0.17, *se* = 0.06, β = 0.26, *p* = 0.005), and vice versa (*b* = 0.24, *se* = 0.10, β = 0.15, *p* = 0.007); however, within-person change in self-objectification was not associated with prior or subsequent changes in depressive symptoms. At the between-person level, the random intercept of self-objectification was significantly correlated with ASMC (*r* = 0.75, *b* = 0.50, *se* = 0.11, *p* < 0.001) and depressive symptoms (*r* = 0.56, *b* = 0.37, *se* = 0.09, *p* < 0.001).

### Sensitivity Analyses: Estimation and Sample

Next, two models were estimated in which the depressive symptoms variables were modeled as ordinal categorical variables using WLSMV estimation; one model included time on social media as a covariate and one included self-objectification. Then, the original model was re-run, limiting the analytic sample to adolescents who reported ever using social media (*n* = 1488). Finally, the original model was re-run, limiting the analytic sample to adolescents who participated at all waves (*n* = 874) to ensure that missing data procedures were not biasing results. In all cases, there were no differences in the pattern of results (in terms of directionality or statistical significance of between-person random intercept correlations and within-person autoregressive and cross-lagged parameters). Thus, the original results were considered robust to these additional specifications.

## Discussion

Given mixed evidence regarding the role of social media use in adolescent depression, research is needed that examines specific, subjective experiences with social media that may confer risk for depressive symptoms across development. In many studies, appearance-related social media experiences have emerged as stronger predictors of depressive symptoms than overall time on social media (Choukas-Bradley et al., 2022). Recent research identified appearance-related social media consciousness at baseline as a predictor of depressive symptoms in middle to late adolescence at a one-year follow-up (Maheux et al., 2022a). However, little is known about how within-person change in appearance-related social media experiences may affect within-person change in depressive symptoms over time. Additionally, most studies have focused on late adolescence and emerging adulthood. The present study addressed these gaps in the literature by investigating within-person associations between appearance-related social media consciousness and depressive symptoms in a large, diverse sample of U.S. youth during early-to-middle adolescence. Hypotheses stated that within-person change in appearance-related social media consciousness would predict subsequent within-person change in depressive symptoms across one year, and this hypothesis was supported, even in the context of controlling for time on social media and offline self-objectification. Importantly, evidence did not suggest the reverse temporal ordering: change in depressive symptoms was not associated with subsequent change in appearance-related social media consciousness. Models also examined the role of gender on an exploratory basis, finding that although girls reported higher mean levels of appearance-related social media consciousness and depressive symptoms, within-person associations between appearance-related social media consciousness and depressive symptoms were similar across boys and girls, suggesting no differential risk associated with heightened appearance-related social media consciousness across these groups. Overall, these results suggest that the highly visual, appearance-focused nature of social media, paired with heightened risk for appearance concerns during adolescence, may exacerbate early and middle adolescents’ vulnerability to depressive symptoms.

### ASMC and Subjective Social Media Experiences

In the current study, young adolescents’ within-person increases in appearance-related social media consciousness were linked with subsequent within-person increases in depressive symptoms. Previous cross-sectional (Choukas-Bradley et al., 2020) and between-person longitudinal work (Maheux et al., 2022a) with middle and late adolescents (roughly ages 14 to 18), found that appearance-related social media consciousness is predictive of adolescents’ depressive symptoms. Specifically, longitudinal research across two waves of data collection with U.S. high school students found that between-person levels of appearance-related social media consciousness preceded increased depressive symptoms, but not vice versa (Maheux et al., 2022a). Although many offline and online experiences may be bidirectionally associated across cascading developmental systems (Flannery et al., 2023), and research indicates that adolescents with depression tend to use social media in distinct ways (Radovic et al., 2017), prior and current results provide growing evidence that online appearance concerns precede depressive symptoms. Cognitive (Beck, 2002) and interpersonal factors (Rudolph et al., 2008) facilitate the onset and maintenance of depressive symptoms, supporting the importance of subjective, appearance-related social media experiences in depressive symptomatology. The current results are also aligned with recent theoretical work positing that appearance concerns may be a key, underexplored mechanism linking social media use to depressive symptoms (Choukas-Bradley et al., 2022).

Moreover, the current study supports and extends prior work by identifying patterns of intra-individual developmental processes among a younger sample. Early adolescence may be a unique risk period, when youth may be more sensitive to social media content (Orben et al., 2022), such as social rewards and evaluative concerns regarding self-presentation. Research has not yet systematically investigated the role of appearance-related social media consciousness in mental health across the lifespan. However, prior research has reported bivariate correlations between appearance-related social media consciousness and depressive symptoms of *r* = 0.30–0.33 among young adults (Maheux et al., 2022b) and, among high school-aged adolescents, *r* = 0.37 for girls and *r* = 0.26 for boys (Maheux et al., 2022a). In the current study, cross-sectional correlations of *r* = 0.46–0.51 for girls and *r* = 0.33–0.36 for boys suggest that younger adolescents may be more sensitive to the negative effects of appearance-related social media consciousness, although future longitudinal and cohort studies should directly assess these possibilities. Additionally, the analytic approach of the current study allows for the disaggregation of between- and within-person effects (see Curran & Bauer, 2011) to identify lead-lag relationships (i.e., temporal ordering) between changes in appearance-related social media consciousness and depressive symptoms. To date, this approach has been mostly absent from the literature on adolescents’ appearance-related social media use and mental health, despite the possibility that associations at different levels of analysis may be reversed (i.e., “Simpson’s Paradox”; Kievit et al., 2013). The current approach is critical to identify—and potentially intervene on—change processes happening *within* an individual over the course of development. Although some prior work has shown that within-person change in appearance-oriented social media use, including posting appearance content (Schreurs & Vandenbosch, 2022) and liking or commenting on others’ posts (Steinsbekk et al., 2021), is associated with subsequent within-person change in body esteem, the current study extends this work by linking appearance-oriented social media use directly with depressive symptoms.

The current study also assessed how within-person processes may differ between individuals based on gender. Although girls were more likely to report heightened appearance-related social media consciousness, within-person associations with depressive symptoms were similar among boys and girls. These findings are consistent with prior work finding higher levels of appearance concerns on social media among girls relative to boys, but not necessarily higher risk of negative outcomes resulting from such appearance concerns. Specifically, prior work on appearance-related social media consciousness has shown these patterns cross-sectionally (Choukas-Bradley et al., 2020) and longitudinally (Maheux et al., 2022a) during middle-to-late adolescence. Other work measuring appearance preoccupation (Hawes et al., 2020) and highly-visual social media use (Jarman et al., 2021) has found similar patterns. In other words, research increasingly supports a main effect of gender predicting both appearance concerns and depressive symptoms, but not moderation by gender. The body of research on social media and appearance concerns focuses disproportionately on girls and women (see Choukas-Bradley et al., 2022), but masculine appearance ideals presented on social media, such as muscularity (Gültzow et al., 2020), may encourage appearance concerns among boys (Rodgers et al., 2020). Thus, a particular focus in future research on social media-related appearance concerns and mental health among adolescent boys is warranted.

Critically, results from sensitivity analyses highlight that the association between appearance-related social media consciousness and later depressive symptoms holds when controlling for time on social media and self-objectification. These results are consistent with past cross-sectional work, which found that higher appearance-related social media consciousness was associated with higher depressive and disordered eating symptoms, controlling for time on social media, gender, race/ethnicity, and body surveillance (Choukas-Bradley et al., 2020). Longitudinal work with middle adolescents also found that ASMC is associated with increases in depressive symptoms, controlling for gender and time on social media (Maheux et al., 2022a). Moreover, within-person links between changes in depressive symptoms and either time on social media or self-objectification were not significant in the context of controlling for appearance-related social media consciousness, indicating the likely stronger role of appearance-related social media consciousness in predicting change in depressive symptoms. Notably, results indicated that, at the between-person level, appearance-related social media consciousness is associated with both time on social media and self-objectification; thus, youth who use social media more and engage in more offline self-objectification may be more likely to engage in appearance preoccupation online overall, and perhaps subsequently experience depressive symptoms. However, time on social media was not associated with depressive symptoms at the between- or within-person levels, further supporting calls to focus on specific subjective experiences rather than screen time (Odgers & Jensen, 2020). The possibility that offline and online appearance concerns may be mutually reinforcing, suggested by the cross-lagged results between appearance-related social media consciousness and self-objectification, is an important area of future inquiry. Future research should systematically address how different aspects of body image—including appearance-related social media consciousness, body dissatisfaction, and self-objectification—may correlate and differentially relate to mental health.

### Implications

Much of the research on social media use and mental health considers adolescence as a monolithic developmental period. The current study sampled adolescents in the early and middle adolescence periods, a time when social media use often begins (Rideout et al., 2022). Recent evidence suggests that windows of heightened sensitivity to media effects occur roughly around puberty for adolescents (i.e., 11–13 among girls and 14–15 among boys; Orben et al., 2022). Puberty, the initiation of dating relationships, heightened concern about peer approval, and intensified self-consciousness (Dahl et al., 2018) all contribute to early and middle adolescents’ appearance concerns and depression (Sharpe et al., 2018). Appearance-related experiences on social media may influence these normative developmental cascades (Flannery et al., 2023), as the accumulating effects of adolescent developmental changes, sociocultural expectations about appearance, and features of social media that make appearance salient may progressively increase the risk for mental health concerns (Choukas-Bradley et al., 2022). The present results underscore the role of social media-specific appearance concerns as a putative contributor to elevated depressive symptoms in U.S. middle school students today. Although increases in body image concerns are normative during this period (Markey, 2010), mental health challenges experienced during this vulnerable time can affect later adolescent and adult well-being (Blashill & Wilhelm, 2014). Guided by the differential susceptibility to media effects model (Valkenburg & Peter, 2013), future work can investigate between-person differences in within-person couplings between change in appearance-related social media consciousness and depressive symptoms that unfold across the lifespan—from late childhood to early adulthood.

The current results also have theoretical and translational implications. Results support the growing consensus that researchers studying social media use and depression in the digital era should measure subjective social media experiences, rather than simplistic measures of “screen time” (Odgers & Jensen, 2020). Basic research investigating inter-individual factors influencing intra-individual change during childhood and early adolescence could contribute to earlier detection and prevention of depression; for example, future work should identify who may be at highest risk of elevated appearance-related social media consciousness, perhaps based on social identity, rejection sensitivity, or offline contextual factors. Interventions targeting negative social media-related mental health outcomes could focus on appearance-related cognitions and behaviors, rather than solely aiming to reduce adolescents’ time using social media. For example, social media literacy interventions have been successful in reducing body image-related mental health outcomes (Gordon et al., 2021) and could specifically target appearance-related social media consciousness to potentially mitigate the development of depression. Finally, current U.S. policy proposals emphasize enforcing age restrictions on social media and limiting features that encourage problematic or habitual use. The current results support the need for such policies and may suggest the need for more stringent limitations to features that exacerbate appearance-related social media consciousness and appearance-focused use, such as likes, photo-editing, and algorithms that prioritize user engagement over other factors.

### Limitations and Future Directions

Several limitations should be noted, including that data were collected from a single school district. Results may not generalize to early and middle adolescents across the U.S. or elsewhere. Additionally, the group of gender minority youth was not large enough to include in comparative analyses. Future work should examine the role of adolescents’ diverse identities and developmental contexts in social media experiences, given recent results suggesting that gender minority adolescents report unique benefits related to using social media and curating online feeds (Coyne et al., 2023) that may be related to viewing affirming appearance imagery. This study also used exclusively self-report data, which may have led to misreporting (Scharkow, 2016). However, subjective and intrapersonal social media experiences, such as appearance-related social media consciousness, are likely best captured with self-reports. Future work can incorporate multiple methodologies to bolster self-report measures, such as using eye-tracking as a biobehavioral marker of appearance-related social media consciousness while participants use social media. The measure of appearance-related social media consciousness also asks specifically about social media photos, rather than videos. In the years since the development of the Appearance-Related Social Media Consciousness Scale, social media videos have become increasingly popular through the use of TikTok (Rideout et al., 2022) and should be examined in future work.

Additionally, although our analyses provided the first examination of temporal ordering of within-person associations in these constructs, they should not be misconstrued for evidence of causality. Multiple large-scale replications of these longitudinal results and future experimental work can collectively provide the best evidence of causal pathways between appearance-related social media experiences and mental health. Future longitudinal work should also examine links between appearance-related social media consciousness and other problematic outcomes, such as anxiety symptoms. Finally, data collection for the current study occurred during the COVID-19 pandemic. It is possible that for some youth, online body image concerns and depressive symptoms increased during this time, when peer interactions were almost entirely mediated by digital channels (Hamilton et al., 2022); thus, replication is needed to address if these results are generalizable to other historical contexts.

## Conclusion

Prior research has examined the role of appearance-related social media consciousness in adolescents’ depressive symptoms, yet mostly focused on between-person differences and youth in middle or late adolescence. The current study extends this work by examining within-person change in appearance-related social media consciousness and depressive symptoms during early adolescence. Results indicate that within-person increases in appearance-related social media consciousness precede increases in depressive symptoms, rather than vice versa, indicating that young adolescents who experience more appearance-related social media consciousness than they usually do may subsequently report heightened depressive symptoms. Given that no differences were found across gender, results highlight troubling patterns of developmental cascades, in which those who become increasingly preoccupied with their online self-presentation may be at risk for negative mental health in the digital era. Future work should build on these findings by continuing to investigate the role of subjective social media experiences—rather than time on social media—in adolescent mental health, and should inform the rapid development of policy solutions to these societal challenges.

## Supplementary Information

Supplementary Information

## References

[CR1] Beck, A. T. (2002). Cognitive models of depression. *Clinical advances in cognitive psychotherapy: Theory and Application*, *14*(1), 29–61.

[CR72] Blashill, A. J., & Wilhelm, S. (2014). Body image distortions, weight, and depression in adolescent boys: Longitudinal trajectories into adulthood. *Psychology of Men & Masculinity, 15*(4), 445–451. 10.1037/a0034618. 10.1037/a0034618 PMC4219600 25383047

[CR2] Bollen, K. A. (1989). *Structural equations with latent variables*. John Wiley & Sons. 10.1002/9781118619179.

[CR67] Choukas-Bradley, S., Nesi, J., Widman, L., & Higgins, M. K. (2019). Camera-ready: Young women’s appearance-related social media consciousness. *Psychology of Popular Media Culture*, *8*(4), 473–481. 10.1037/ppm0000196.

[CR5] Choukas-Bradley, S., Nesi, J., Widman, L., & Galla, B. M. (2020). The Appearance-Related Social Media Consciousness Scale: Development and validation with adolescents. *Body Image*, *33*, 164–174. 10.1016/j.bodyim.2020.02.017. 32193170 10.1016/j.bodyim.2020.02.017

[CR6] Choukas-Bradley, S., Maheux, A. J., Roberts, S. R., & Nesi, J. (2022). The perfect storm: A developmental–sociocultural framework for the role of social media in adolescent girls’ body image concerns and mental health. *Clinical Child and Family Psychology Review*, *25*, 681–701. 10.1007/s10567-022-00404-5. 35841501 10.1007/s10567-022-00404-5 PMC9287711

[CR7] Coyne, S. M., Weinstein, E., Sheppard, J. A., James, S., Gale, M., Van Alfen, M., Ririe, N., Monson, C., Ashby, S., Weston, A., & Banks, K. (2023). Analysis of social media use, mental health, and gender identity among US youths. *JAMA Network Open*, *6*(7), e2324389. 10.1001/jamanetworkopen.2023.24389. 37486631 10.1001/jamanetworkopen.2023.24389 PMC10366700

[CR8] Crone, E., & Dahl, R. (2012). Understanding adolescence as a period of social–affective engagement and goal flexibility. *Nature Reviews Neuroscience*, *13*, 636–650. 10.1038/nrn3313. 22903221 10.1038/nrn3313

[CR9] Curran, P. J., & Bauer, D. J. (2011). The disaggregation of within-person and between-person effects in longitudinal models of change. *Annual Review of Psychology*, *62*, 583–619. 10.1146/annurev.psych.093008.100356. 19575624 10.1146/annurev.psych.093008.100356 PMC3059070

[CR10] Dahl, R. E., Allen, N. B., Wilbrecht, L., & Suleiman, A. B. (2018). Importance of investing in adolescence from a developmental science perspective. *Nature*, *554*(7693), 441–450. 10.1038/nature25770. 29469094 10.1038/nature25770

[CR11] Daniels, E. A., Zurbriggen, E. L., & Monique Ward, L. (2020). Becoming an object: A review of self-objectification in girls. *Body Image*, *33*, 278–299. 10.1016/j.bodyim.2020.02.016. 32470822 10.1016/j.bodyim.2020.02.016

[CR12] DeCoster, J., Gallucci, M., & Iselin, A.-M. R. (2011). Best practices for using median splits, artificial categorization, and their continuous alternatives. *Journal of Experimental Psychopathology*, *2*(2), 197–209. 10.5127/jep.008310.

[CR14] Ferreiro, F., Seoane, G., & Senra, C. (2014). Toward understanding the role of body dissatisfaction in the gender differences in depressive symptoms and disordered eating: A longitudinal study during adolescence. *Journal of Adolescence*, *37*(1), 73–84. 10.1016/j.adolescence.2013.10.013. 24331307 10.1016/j.adolescence.2013.10.013

[CR71] Flannery, J. S., Maza, M. T., Kilic, Z., & Telzer, E. H. (2023). Cascading bidirectional influences of digital media use and mental health in adolescence. In Lockman, J. J. (Ed.) Advances in Child Development and Behavior (Vol. 64, pp. 255–287). Elsevier. 10.1016/bs.acdb.2022.10.003. 10.1016/bs.acdb.2022.10.003 37080671

[CR15] Fredrickson, B. L., & Roberts, T. (1997). Objectification theory: Toward understanding women’s lived experiences and mental health risks. *Psychology of Women Quarterly*, *21*, 173–206. 10.1111/j.1471-6402.1997.tb00108.x.

[CR16] Galanaki, E. P. (2012). The imaginary audience and the personal fable: A test of Elkind’s Theory of Adolescent Egocentrism. *Psychology*, *3*(6), 457–466. 10.4236/psych.2012.36065.

[CR17] Geiger, A. W., & Davis, L. (2019). A growing number of American teenagers – particularly girls – are facing depression. *Pew Research Center*. https://www.pewresearch.org/fact-tank/2019/07/12/.

[CR18] Gordon, C. S., Jarman, H. K., Rodgers, R. F., McLean, S. A., Slater, A., Fuller-Tyszkiewicz, M., & Paxton, S. J. (2021). Outcomes of a cluster randomized controlled trial of the SoMe social media literacy program for improving body image-related outcomes in adolescent boys and girls. *Nutrients*, *13*(11), 3825. 10.3390/nu13113825. 34836084 10.3390/nu13113825 PMC8674763

[CR20] Gültzow, T., Guidry, J. P. D., Schneider, F., & Hoving, C. (2020). Male body image portrayals on Instagram. *Cyberpsychology, Behavior, and Social Networking*, *23*(5), 281–289. 10.1089/cyber.2019.0368. 32286866 10.1089/cyber.2019.0368

[CR21] Ha, T., Overbeek, G., & Engels, R. C. M. E. (2010). Effects of attractiveness and social status on dating desire in heterosexual adolescents: An experimental study. *Archives of Sexual Behavior*, *39*(5), 1063–1071. 10.1007/s10508-009-9561-z. 19830538 10.1007/s10508-009-9561-z PMC2933005

[CR22] Hamaker, E. L., Kuiper, R. M., & Grasman, R. P. P. P. (2015). A critique of the cross-lagged panel model. *Psychological Methods*, *20*(1), 102–116. 10.1037/a0038889. 25822208 10.1037/a0038889

[CR23] Hamilton, J. L., Nesi, J., & Choukas-Bradley, S. (2022). Reexamining social media and socioemotional well-being among adolescents through the lens of the COVID-19 pandemic: A theoretical review and directions for future research. *Perspectives on Psychological Science*, *17*(3), 662–679. 10.1177/17456916211014189. 34756118 10.1177/17456916211014189 PMC9081105

[CR24] Hawes, T., Zimmer-Gembeck, M. J., & Campbell, S. M. (2020). Unique associations of social media use and online appearance preoccupation with depression, anxiety, and appearance rejection sensitivity. *Body Image*, *33*, 66–76. 10.1016/j.bodyim.2020.02.010. 32113009 10.1016/j.bodyim.2020.02.010

[CR26] Jarman, H. K., Marques, M. D., McLean, S. A., Slater, A., & Paxton, S. J. (2021). Motivations for social media use: Associations with social media engagement and body satisfaction and well-being among adolescents. *Journal of Youth and Adolescence*. 10.1007/s10964-020-01390-z. 10.1007/s10964-020-01390-z 33475925

[CR28] Jones, D. C., Vigfusdottir, T. H., & Lee, Y. (2004). Body image and the appearance culture among adolescent girls and boys: An examination of friend conversations, peer criticism, appearance magazines, and the internalization of appearance ideals. *Journal of Adolescent Research*, *19*(3), 323–339. 10.1177/0743558403258847.

[CR29] Kievit, R. A., Frankenhuis, W. R., Waldorp, L. J., & Borsboom, D. (2013). Simpson’s paradox in psychological science: A practical guide. *Frontiers in Psychology*, *4*(513). 10.3389/fpsyg.2013.00513. 10.3389/fpsyg.2013.00513 PMC3740239 23964259

[CR30] Lindner, D., & Tantleff-Dunn, S. (2017). The Development and Psychometric Evaluation of the Self-Objectification Beliefs and Behaviors Scale. *Psychology of Women Quarterly*, *41*(2), 254–272. 10.1177/0361684317692109.

[CR31] Maheux, A. J., Roberts, S. R., Nesi, J., Widman, L., & Choukas-Bradley, S. (2022b). Psychometric properties and factor structure of the Appearance-Related Social Media Consciousness Scale among emerging adults. *Body Image*, *43*, 63–74. 10.1016/j.bodyim.2022.08.002. 36055008 10.1016/j.bodyim.2022.08.002 PMC10224750

[CR32] Maheux, A. J., Roberts, S. R., Nesi, J., Widman, L., & Choukas-Bradley, S. (2022a). Longitudinal associations between appearance-related social media consciousness and adolescents’ depressive symptoms. *Journal of Adolescence*, *94*(2), 264–269. 10.1002/jad.12009. 10.1002/jad.12009 PMC8974358 35353426

[CR34] Markey, C. N. (2010). Invited commentary: Why body image is important to adolescent development. *Journal of Youth and Adolescence*, *39*, 1387–1391. 10.1007/s10964-010-9510-0. 20339908 10.1007/s10964-010-9510-0

[CR35] Maughan, B., Collishaw, S., & Stringaris, A. (2013). Depression in childhood and adolescence. *Journal of the Canadian Academy of Child and Adolescent Psychiatry*, *22*(1), 35–40. 10.1080/1047840X.2020.1820214. 23390431 PMC3565713

[CR36] Mayeux, L., & Kleiser, M. (2020). A gender prototypicality theory of adolescent peer popularity. *Adolescent Research Review*, *5*(3), 295–306. 10.1007/s40894-019-00123-z.

[CR37] Mulder, J. D., & Hamaker, E. L. (2021). Three extensions of the random intercept cross-lagged panel model. *Structural Equation Modeling: A Multidisciplinary Journal*, *28*(4), 638–648. 10.1080/10705511.2020.1784738.

[CR38] Muthén, B. (2022). Mplus web talk no. 4 – using Mplus to do cross-lagged modeling of panel data part 2: categorical variables. *Statmodel.com*. https://www.statmodel.com/Webtalk4P2.shtml.

[CR39] Muthén, B., & Muthén, L. (2017). *Mplus User’s Guide*. 8th ed. Los Angeles, CA: Muthén & Muthén.

[CR40] Nesi, J., & Prinstein, M. J. (2015). Using social media for social comparison and feedback-seeking: gender and popularity moderate associations with depressive symptoms. *Journal of Abnormal Child Psychology*, *43*(8), 1427–1438. 10.1007/s10802-015-0020-0. 25899879 10.1007/s10802-015-0020-0 PMC5985443

[CR41] Nesi, J., Choukas-Bradley, S., & Prinstein, M. J. (2018). Transformation of adolescent peer relations in the social media context: Part 1—A theoretical framework and application to dyadic peer relationships. *Clinical Child and Family Psychology Review*, *21*(3), 267–294. 10.1007/s10567-018-0261-x. 29627907 10.1007/s10567-018-0261-x PMC6435354

[CR42] Nesi, J., Choukas-Bradley, S., Maheux, A. J., Roberts, S. R., Sanzari, C., Widman, L., & Prinstein, M. J. (2021). Selfie appearance investment and peer feedback concern: Multi-method investigation of adolescent selfie practices and psychosocial adjustment. *Psychology of Popular Media*, *10*(4), 488–499. 10.1037/ppm0000342. 10.1037/ppm0000342 PMC8735707 35003884

[CR43] Odgers, C. L., & Jensen, M. R. (2020). Annual research review: Adolescent mental health in the digital age: Facts, fears, and future directions. *Journal of Child Psychology and Psychiatry*, *61*(3), 336–348. 10.1111/jcpp.13190. 31951670 10.1111/jcpp.13190 PMC8221420

[CR44] Orben, A. (2020). Teenagers, screens and social media: A narrative review of reviews and key studies. *Social Psychiatry and Psychiatric Epidemiology*, *55*(4), 407–414. 10.1007/s00127-019-01825-4. 31925481 10.1007/s00127-019-01825-4

[CR45] Orben, A., Przybylski, A. K., Blakemore, S. J., & Kievit, R. A. (2022). Windows of developmental sensitivity to social media. *Nature Communications*, *13*, 1649. 10.1038/s41467-022-29296-3. 35347142 10.1038/s41467-022-29296-3 PMC8960761

[CR46] Özparlak, A., & Karakaya, D. (2022). The associations of cognitive distortions with internet addiction and internet activities in adolescents: A cross-sectional study. *Journal of Child and Adolescent Psychiatric Nursing*, *35*(4), 322–330. 10.1111/jacp.12385. 35637598 10.1111/jcap.12385

[CR49] R Core Team. (2021). *R: A language and environment for statistical computing*. R Foundation for Statistical Computing, Vienna, Austria. https://www.R-project.org/.

[CR50] Radovic, A., Gmelin, T., Stein, B. D., & Miller, E. (2017). Depressed adolescents’ positive and negative use of social media. *Journal of Adolescence*, *66*, 5–15. 10.1016/j.adolescence.2016.12.002. 10.1016/j.adolescence.2016.12.002 PMC5485251 27997851

[CR51] Rideout, V., Peebles, A., Mann, S., & Robb, M. B. (2022). Common Sense census: Media use by tweens and teens, 2021. Common Sense. https://www.commonsensemedia.org/research/the-common-sense-census-media-use-by-tweens-and-teens-2021.

[CR52] Ridolfi, D. R., Myers, T. A., Crowther, J. H., & Ciesla, J. A. (2011). Do appearance focused cognitive distortions moderate the relationship between social comparisons to peers and media images and body image disturbance? *Sex Roles*, *65*(7), 491. 10.1007/s11199-011-9961-0.

[CR53] Rodgers, R. F., & Melioli, T. (2016). The relationship between body image concerns, eating disorders and internet use, part I: A review of empirical support. *Adolescent Research Review*, *1*(2), 95–119. 10.1007/s40894-015-0016-6.

[CR54] Rodgers, R. F., Slater, A., Gordon, C. S., McLean, S. A., Jarman, H. K., & Paxton, S. J. (2020). A biopsychosocial model of social media use and body image concerns, disordered eating, and muscle-building behaviors among adolescent girls and boys. *Journal of Youth and Adolescence*, *49*(2), 399–409. 10.1007/s10964-019-01190-0. 31907699 10.1007/s10964-019-01190-0

[CR68] Rogol, A. D., Roemmich, J. N., & Clark, P. A. (2002). Growth at puberty. *Journal of Adolescent Health, 31*(6), 192–200. 10.1016/S1054-139X(02)00485-8. 10.1016/s1054-139x(02)00485-8 12470915

[CR55] Rudolph, K. D., Flynn, M., & Abaied, J. L. (2008). A developmental perspective on interpersonal theories of youth depression. In J. R. Z. Abela & B. L. Hankin (Eds.), *Handbook of depression in children and adolescents* (pp. 79–102, Chapter xii, 529 Pages). The Guilford Press.

[CR70] Satorra, A., & Bentler, P. M. (2001). A scaled difference chi-square test statistic for moment structure analysis. *Psychometrika 66*, 507–514. 10.1007/BF02296192 10.1007/s11336-009-9135-y PMC2905175 20640194

[CR57] Scharkow, M. (2016). The accuracy of self-reported internet use—A validation study using client log data. *Communication Methods and Measures*, *10*(1), 13–27. 10.1080/19312458.2015.1118446.

[CR58] Schreurs, L., & Vandenbosch, L. (2022). Different interactions with appearance-focused social media content and adolescents’ body dissatisfaction: A within-person perspective. *Computers in Human Behavior*, *135*. 10.1016/j.chb.2022.1107364.

[CR59] Sharp, C., Goodyer, I. M., & Croudace, T. J. (2006). The Short Mood and Feelings Questionnaire (SMFQ): A unidimensional item response theory and categorical data factor analysis of self-report ratings from a community sample of 7-through 11-year-old children. *Journal of Abnormal Child Psychology*, *34*(3), 365–377. 10.1007/s10802-006-9027-x. 10.1007/s10802-006-9027-x 16649000

[CR60] Sharpe, H., Patalay, P., Choo, T.-H., Wall, M., Mason, S. M., Goldschmidt, A. B., & Neumark-Sztainer, D. (2018). Bidirectional associations between body dissatisfaction and depressive symptoms from adolescence through early adulthood. *Development and Psychopathology*, *30*(4), 1447–1458. 10.1017/S0954579417001663. 29144209 10.1017/S0954579417001663 PMC6343674

[CR61] Slater, A., & Tiggemann, M. (2015). Media exposure, extracurricular activities, and appearance-related comments as predictors of female adolescents’ self-objectification. *Psychology of Women Quarterly*, *39*(3), 375–389. 10.1177/0361684314554606.

[CR62] Steinsbekk, S., Wichstrøm, L., Stenseng, F., Nesi, J., Hygen, B. W., & Skalická, V. (2021). The impact of social media use on appearance self-esteem from childhood to adolescence – A 3-wave community study. *Computers in Human Behavior*, *114*, 106528. 10.1016/j.chb.2020.106528.

[CR63] Terán, L., Yan, K., & Aubrey, J. S. (2020). “But first let me take a selfie”: U.S. adolescent girls’ selfie activities, self-objectification, imaginary audience beliefs, and appearance concerns. *Journal of Children and Media*, *14*(3), 343–360. 10.1080/17482798.2019.1697319.

[CR64] Turner, N., Joinson, C., Peters, T. J., Wiles, N., & Lewis, G. (2014). Validity of the Short Mood and Feelings Questionnaire in late adolescence. *Psychological Assessment*, *26*(3), 752–762. 10.1037/a0036572. 24749755 10.1037/a0036572

[CR69] Twenge, J. M., Joiner, T. E., Rogers, M. L., & Martin, G. N. (2018). Increases in depressive symptoms, suicide related outcomes, and suicide rates among U.S. adolescents after 2010 and links to increased new media screen time. *Clinical Psychological Science, 6*(1), 3–17. 10.1177/2167702617723376

[CR65] Valkenburg, P. M., & Peter, J. (2013). The differential susceptibility to media effects model. *Journal of Communication*, *63*(2), 221–243. 10.1111/jcom.12024.

