# Supplementary material for: Longitudinal Change in Appearance-Related Social Media Consciousness and Depressive Symptoms: A Within-Person Analysis during Early-to-Middle Adolescence
Source: J Youth Adolesc. 2024 May 24;53(10):2287–99. doi: 10.1007/s10964-024-01998-5 (PMC11413103; doi:10.1007/s10964-024-01998-5)
Supplement: Supplementary file 1 — Supplementary Information [file 10964_2024_1998_MOESM1_ESM.docx]

| **Supplemental Table 1**  *Within-Person Autoregressive and Cross-Lagged Paths and Within-Wave Covariances from the Random Intercept Cross-Lagged Panel Model Controlling for Time on SM* | | | | | |
| --- | --- | --- | --- | --- | --- |
|  |  | Full sample (*n* = 1594) | | | |
|  |  | *b* | *SE* | β | *p* |
| Autoregressive (Lagged) Paths | |  |  |  |  |
|  | ASMC 🡪 ASMC | 0.59 | 0.13 | .57, .59 | < .001 |
|  | DS 🡪 DS | 0.39 | 0.09 | .37 | < .001 |
|  | SM Time 🡪 SM Time | 0.27 | 0.10 | .28, .29 | 0.005 |
| Cross-Lagged Paths | |  |  |  |  |
|  | ASMC 🡪 DS | 0.24 | 0.08 | .23, .24 | .003 |
|  | DS 🡪 ASMC | 0.10 | 0.06 | .09, .10 | .108 |
|  | DS 🡪 SM Time | 0.24 | 0.14 | .12, .14 | .097 |
|  | ASMC 🡪 SM Time | 0.24 | 0.16 | .13, .14 | .146 |
|  | SM Time 🡪 ASMC | 0.02 | 0.03 | .04 | .466 |
|  | SM Time 🡪 DS | 0.04 | 0.03 | .07, .08 | .161 |
| Within-Person Within-Wave Covariances | |  |  |  |  |
|  | ASMC T1 with DS T1 | .48 | .20 | .50 | .015 |
|  | ASMC T2 with DS T2 | .21 | .04 | .31 | <.001 |
|  | ASMC T3 with DS T3 | .21 | .05 | .29 | <.001 |
|  | ASMC T1 with SM Time T1 | .61 | .28 | .30 | .031 |
|  | ASMC T2 with SM Time T2 | .28 | .11 | .20 | .010 |
|  | ASMC T3 with SM Time T3 | .10 | .08 | .08 | .220 |
|  | SM Time T1 with DS T1 | .60 | .25 | .31 | .018 |
|  | SM Time T2 with DS T2 | .18 | .10 | .13 | .065 |
|  | SM Time T3 with DS T3 | .20 | .10 | .14 | .036 |
| *Note.* ASMC = appearance-related social media consciousness. DS = depressive symptoms. SM = Social media. Cross-lagged and autoregressive paths are constrained to be equal across time. Some standardized parameters vary over time for constrained paths due to different variances. | | | | | |

| **Supplemental Table 2**  *Within-Person Autoregressive and Cross-Lagged Paths and Within-Wave Covariances from the Random Intercept Cross-Lagged Panel Model Controlling for Self-Objectification* | | | | | |
| --- | --- | --- | --- | --- | --- |
|  |  | Full sample (*n* = 1594) | | | |
|  |  | *b* | *SE* | β | *p* |
| Autoregressive (Lagged) Paths | |  |  |  |  |
|  | ASMC 🡪 ASMC | .50 | .13 | .48, .51 | <.001 |
|  | DS 🡪 DS | .39 | .09 | .36, .37 | <.001 |
|  | SO 🡪 SO | .16 | .11 | .16 | .144 |
| Cross-Lagged Paths | |  |  |  |  |
|  | ASMC 🡪 DS | .22 | .09 | .21, .22 | .011 |
|  | DS 🡪 ASMC | .10 | .06 | .09, .10 | .111 |
|  | DS 🡪 SO | .04 | .05 | .06 | .431 |
|  | ASMC 🡪 SO | .17 | .06 | .26 | .005 |
|  | SO 🡪 ASMC | .24 | .09 | .15 | .007 |
|  | SO 🡪 DS | .10 | .10 | .06 | .314 |
| Within-Person Within-Wave Covariances | |  |  |  |  |
|  | ASMC T1 with DS T1 | .46 | .17 | .50 | .007 |
|  | ASMC T2 with DS T2 | .22 | .04 | .32 | <.001 |
|  | ASMC T3 with DS T3 | .20 | .05 | .29 | <.001 |
|  | ASMC T1 with SO T1 | .38 | .11 | .61 | <.001 |
|  | ASMC T2 with SO T2 | .24 | .04 | .50 | <.001 |
|  | ASMC T3 with SO T3 | .17 | .03 | .36 | <.001 |
|  | SO T1 with DS T1 | .23 | .09 | .40 | .009 |
|  | SO T2 with DS T2 | .09 | .03 | .18 | .009 |
|  | SO T3 with DS T3 | .10 | .03 | .19 | .001 |
| *Note.* ASMC = appearance-related social media consciousness. DS = depressive symptoms. SO = self-objectification. Cross-lagged and autoregressive paths are constrained to be equal across time. Some standardized parameters vary over time for constrained paths due to different variances. | | | | | |
